# Comparison of the Response of Male BALB/c and C57BL/6 Mice in Behavioral Tasks to Evaluate Cognitive Function

**DOI:** 10.3390/bs8010014

**Published:** 2018-01-18

**Authors:** Yenela Garcia, Nashelly Esquivel

**Affiliations:** 1 Laboratorio de Antianémicos y Nutracéuticos, Centro Nacional de Biopreparados (BioCen), Carretera Beltrán Km 1 ½, Bejucal 32600, Mayabeque, Cuba; 2 Facultad de Biología, Universidad de la Habana, Plaza de la Revolución 10400, La Habana, Cuba; nesquivel@estudiantes.fbio.uh.cu

**Keywords:** cognitive function, behavioral task, mice

## Abstract

To evaluate several cognitive parameters during the execution of behavioral tasks assessing cognitive function in laboratory animals, the parameters are reported within a range. This situation entails that each laboratory must establish the conditions under which the behavioral task to evaluate the cognitive function can be carried out. C57BL/6 and BALB/c inbred strains are used more often in behavioral studies relating to anxiety, stress, fear and cognitive function. The aim of this work was to compare the behavioral response of mice of the strains BALB/c and C57BL/6 to evaluate memory and learning as cognitive functions. Young male mice, 7–8 weeks of age, from each strain were used. Y maze, object recognition and passive avoidance tasks were performed. Both strains of mice showed differences in the response to the passive avoidance and Y maze task. This study advances knowledge about the baseline behavior of laboratory mice strains and their response during the experimental procedures, which are due to the treatment, genetic influence, procedural differences, genetic background variance, or any combination of these elements.

## 1. Introduction

In recent years, a large number of tests have been developed to evaluate the improvement of cognitive function in laboratory animals that receive different pharmaceutical formulations [1,2,3]. These tests are known, indistinctly, like behavioral models or as behavioral tests. During the development of neuro-pharmaceutical formulations to improve the cognitive function, it becomes indispensable to use combinations of several behavioral tasks to determine their effects and safety. These requirements correspond to the complexity of the cognitive function in humans, in which multiple interconnected brain structures participate [4].

The behavioral tasks that are used to evaluate the cognitive function in laboratory animals include different processes like attention, learning and memory. Learning and memory processes include associative spatial and non-spatial learning and short-term and medium-term memory. Among the spatial memory task, one of the most used is the Morris water maze. Other tests are also used to evaluate spatial and working memory such as the T or Y-maze task sand the radial maze task, as well as non-spatial task of memory, as objects recognition task [5]. Another group of tasks are based on associative learning as is the case of passive avoidance and active avoidance [6].

Species more frequently used as laboratory animals for the execution of behavioral task include rats, mice, non-human primates and non-conventional animals such as the zebra fish [7,8,9]. In the case of mice, the strains C57BL/6 and BALB/c are the most commonly used in behavioral studies relation to stress, fear and cognitive function [10,11,12,13]. During execution of behavioral task to evaluate cognitive function, the parameters used are reported in a range, both, for the test run times and for the times that elapse before returning the animal to the task [3,4,14,15]. This situation entails that each laboratory must establish the conditions under which behavioral task to evaluate the cognitive function can be carried out.

On the other hand, the stress response and strain differences in stress response are one of the most important elements that affect cognitive response [12,13,16,17]. Although, there are several studies in the scientific literature reporting comparisons in the behavioral response in tasks to assess cognitive function in the different inbred strains of mice [11,12,13,16,17,18,19] (Table 1), this aspect remains to be investigated. The aim of this work was to compare the behavioral response of male mice of the strains BALB/c and C57BL/6 for evaluate memory and learning as cognitive functions.

## 2. Materials and Methods

### Animals and Housing Conditions

Young male *Mus musculus* mice (7–8 weeks of age) of the strain BALB/c and C57BL/6 were used, which were supplied by *Centro Nacional para la Producción de Animales de Laboratorio* (CENPALAB). Animals were placed in polypropylene boxes with wood chip bedding (Sournid, Spain). They were kept in a conventional room with controlled environment (type IV barrier of minimum safety), with a temperature of 24 ± 3 °C and relative humidity of 40 to 70%, effecting 18 changes of air per hour, 100% of external air injection with 85% filtration. Circadian rhythms were controlled using a 12 h cycle light/darkness using an automatic timer. Mice were fed the EAO1004_cenp_ diet for rodents ad libitum. Mice were individual identified by ears perforation nomenclature.

An adaptation period of one week prior to the beginning of the experiments was performed. Mice were individually identified by ear perforation and the corporal weight was measured. The experimental protocol was approved by the *Comité Institucional de Ética para eI Cuidado y Uso de Animales de Laboratorio* (CIECUAL). The animals were transferred to the place where the behavioral tests were carried out 24 h before the beginning of the assays. At the end of each task, the surfaces of the mazes were disinfected with a solution of 70% ethanol.

**Figure 1:**
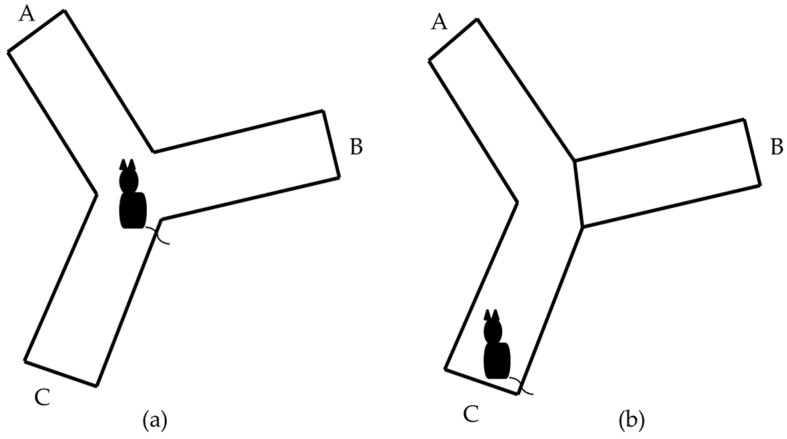
Schematic representation of Y-maze task. The letters A, B and C identify each arm. (**a**) Represents the task with the three open arms; (**b**) Represents the task with one arm blocked.

**Y-maze Task:** it allows evaluating the spatial memory of short-term work and is based on the natural instinct of rodents to explore unknown environments [20]. Y-maze task was used with the three open arms and with one arm blocked. Each task was applied to 12 animals of the BALB/c and C57BL/6 strains. In both cases, an acrylic accessory was used, covered with gray paint, which has three arms of equal length (30 cm), width (10 cm) and height (15 cm), interconnected with each other at a 120° angle. Each arm is identified by a different letter (A, B, C) (Figure 1a).

**Spontaneous alternation:** mice were placed in the center of the Y-maze and during 7 min the animals were allowed to explore the maze freely. A trained experimenter standing in the same position and in silence during the test recorded manually, the sequence of entries in each arm by each mouse. This procedure allowed registering the individual identification of mice, the trial received or experimental group and date. Mice were considered to visit the arm, only when the four limbs passed through the imaginary parallel line originating from the floor of the Y-maze. The animals that made at least 12 entries to the arms were those accepted as valid for the test. In addition, those mice that did not show exploratory activity during the first 2 to 3 min were rejected. The percentage of animals that did not meet the acceptance criteria in the Y-maze task was calculated. The number of alternations was determined with a console application of the Visual Studio program version 2012. Using this application, the sequence of arms visited by each mouse was introduced in the computer that had been registered in the form described above during the execution of the Y-maze task. In the spontaneous alternation task, we considered that the mice made alternations when they sequentially visited the three arms, without repeating any [18]. We also counted the number of repeated entries to the same arm (perseveration) and incorrect entries to the arms for each animal. Finally, spontaneous alternation was calculated by the following expression:

**Spontaneous alternation** (%) = Alternations × 100             Total of Possible Alternations(1)

**Total of Possible Alternations** = Total of entries − 2.

**Y-Maze task (one arm blocked):** The B arm of the Y-maze was blocked and the mouse was placed in the C arm to freely explore C and A arms for 7 min (Figure 1b). Mice did not show exploratory activities during the first 2 to 3 min of the task were rejected. After a period of 30 min, the animal was returned to the task to explore the Y-maze with the three arms free for another 7 min. The behavior of the animal during the second part of the test (three arms free) was recorded with a non-professional digital camera. The percentage of animals that did not meet the acceptance criteria in the Y-maze task (one arm blocked) was calculated. The videos were analyzed in the computer by a trained experimenter to account the total time that the animal visited each arm. The stopwatch tool of window 10 was used for the analysis of the videos. The percentage of time that every animal visited each arm was calculated from the total time of the task by the following expression:

**Percentage of time spent on arms A, B or C (%)** = Time spent visiting arm (s)*×* 100                         Total time to visit the three arms (s)(2)

**Figure 2:**
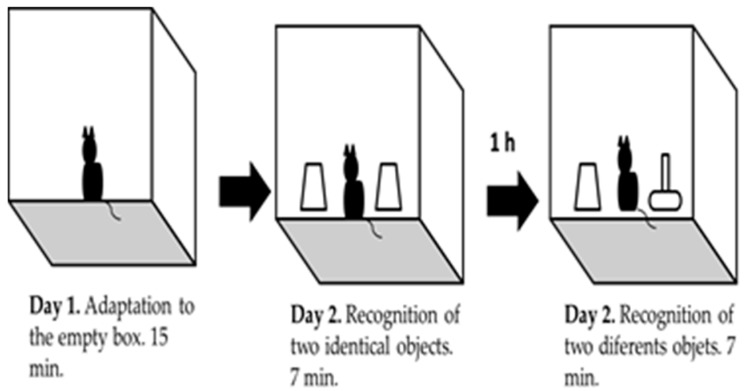
Schematic representation of object recognition task.

**Object recognition task:** it is based on the natural instinct of rodents to explore new objects [15,21]. Prior to the performance of this experimental procedure, we determined that the level of exploration for each pair of different object did not show differences for each strain, as a control measure for source of bias. Twelve animals from each strain were used for this task. The task was done inside an acrylic box of dimensions 22 × 22 × 25 cm and in a first stage of habituation the animal was placed inside the empty box during 15 min (day 1). After 24 h, mice were placed back in the box with two identical objects (Familiar Object, FO). Mice were allowed to explore the objects for 7 min. After 1 h one of the objects was replaced by a novel one (Novel Object, NO) (Figure 2). In this second part of the task, the animal was also allowed to explore both objects for 7 min. Mice’s behavior was recorded with a non-professional digital camera. Animal’s did not show exploratory activities during the first 2 to 3 min of the task were rejected. The percentage of invalid animals was calculated.

The videos were analyzed by a trained experimenter in the computer to account the total time that the animal explored the FO and NO. The stopwatch tool of window 10 was used for the analysis of the videos. It was considered that the animal explored the objects when it bit the object, touched it with the front extremities, nose, or placed the nose at a distance of 2 cm or smaller. In addition, it was not considered an exploration when the animal pushes the object, sits on it, or approaches the object without paying attention. The percent of time that mice spent recognition of the novel object, relative to the total object recognition time or Preference Index (PI), was calculated by the following expression [21]:

PI (%) = [T_NO_/(T_NO_ + T_FO_)] × 100 (3)

**Passive avoidance task:** is based on the innate ability of rodents to recall aversive stimuli and avoid them [3]. Twelve animals from each strain were used for this task. It was used a box with an illuminated chamber (IC) connected to dark chamber (DC) by a guillotine door. In the DC, there is a closed circuit that produces small electric discharges.

**Figure 3:**
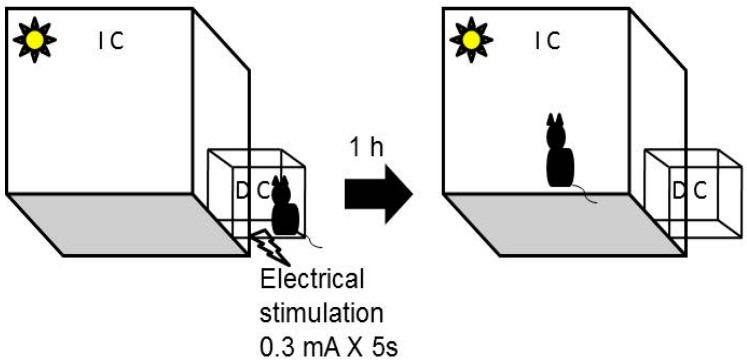
Schematic representation of the passive avoidance task. IC—illuminated chamber. DC—dark chamber.

The task included a first phase (habituation) where the mouse was placed in IC with the guillotine door closed. After 25 s, the guillotine door was opened and the mouse was expected to spontaneously go to it. Mice that in this part of the task did not go to the DC during 180 s or more were rejected. When the mouse spontaneously entered with all four legs in the DC, the guillotine door was closed and it received an electric shock of 0.3 mA during a time of 5 s. The animals were kept inside the DC for 10 s after receiving the electric shock (retention). After 1 h mice came back to the passive avoidance task with free access to both chambers for 300 s (Figure 3). Mice’s behavior was recorded with a non-professional digital camera. The videos were analyzed in the computer by a trained experimenter to account the time that mice delay in entering for the first time into the DC (Latency transition). The stopwatch tool of window 10 was used for the analysis of the videos. Five mice of each strain were used in this task to determine the baseline for transition latency when animal does not receive the electric show in the DC (control mice).

**Statistical analysis:** GraphPad software version 5.0 was used (United States, 2007). All variables were analyzed by the Kolmogorov-Smirnov test to check the normality and variance homogeneity by Fisher’s test. The response of both strains mice to cognitive function tests was compared by a Student’s *t*-test of independent samples or by a one-way ANOVA when more than two samples were analyzed. Tukey’s test was used for the pair group’s comparison. Two-way ANOVA was applied to analyze interaction between the strains and groups (control vs. experimental) in the passive avoidance task. In all cases, a significance level of *p* < 0.05 was used.

## 3. Results

### 3.1. Y-Maze Task

**Figure 4:**
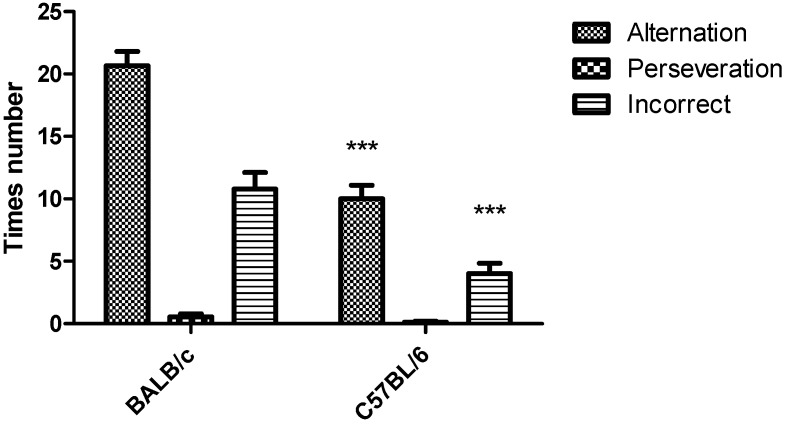
Response to Y-maze task (three arms free) for strains BALB/c and C57BL/6 mice. Data shows mean ± standard deviations for the number of alternation, perseveration or incorrect entries to the arms. * Represents significant differences between the two strains for *p* < 0.05 according to one-way ANOVA and the Tukey *post hoc* test.

**Figure 5:**
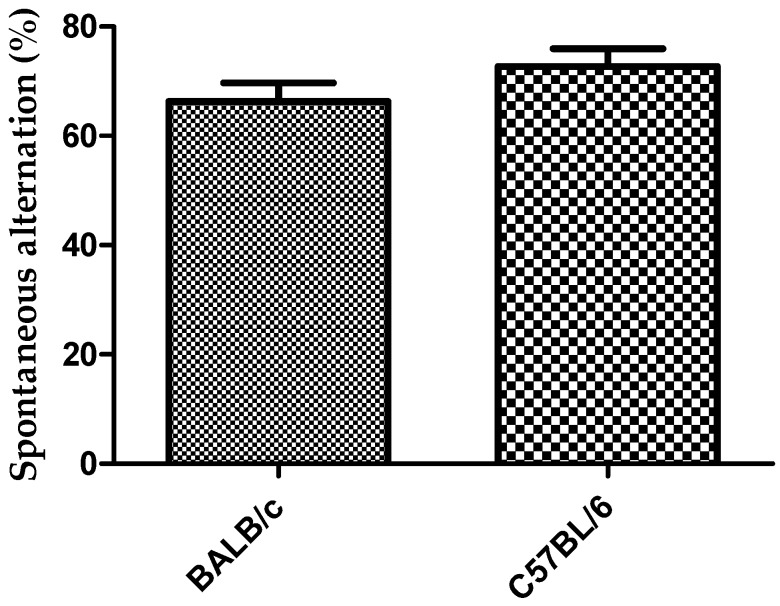
Response to Y-maze task (three arms free) for strains BALB/c and C57BL/6J mice in relation to spontaneous alternation. Data shows mean ± standard deviations for groups.

During spontaneous alternation in Y-test task only one animal of the BALB/c strain was rejected (91% acceptance). Mouse was rejected because it made less than 12 entries to the arms. Two mice of the C57BL/6 strain were rejected (83% acceptance) because the animals did not show exploratory activity during the first 2 to 3 min in the Y-maze task. The number of alternation and incorrect entries to the arms were significantly higher in the BALB/c mice than in C57BL/6 (*p* < 0.001). Number of perseveration did not show significantly differences between the two strains (Figure 4). There was a trend showing that the spontaneous alternation was higher in C57BL/6strain compared to BALB/c, although not statistically significant (Figure 5).

**Figure 6:**
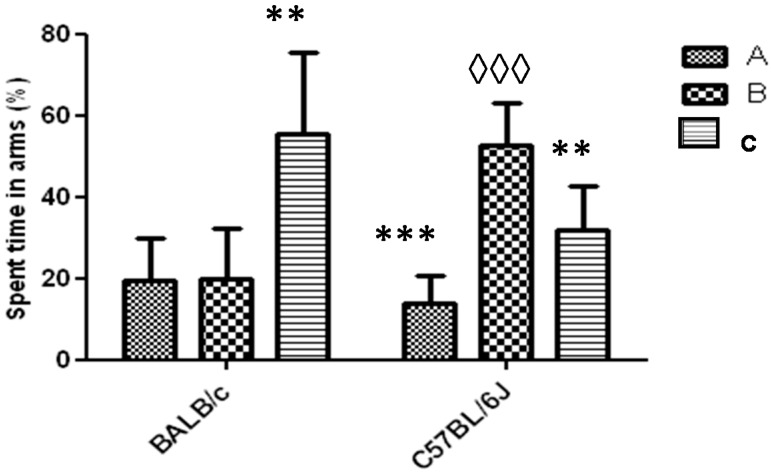
Response to Y-maze task (one arm blocked) for strains BALB/c and C57BL/6J mice. The letters A, B and C identify each arm during the task (B-arm blocked). Data shows mean ± standard deviations for groups in relation to the spent time in each arm. * Represents significant differences with respect to B arm for each strain. ◊ Represents significant differences between the spent times in the blocked arm (B) between the two strains. A one-way ANOVA was used and the Tukey *post hoc* test for *p* < 0.05.

In the Y-Maze task with a blocked arm, mice of the BALB/c strain spent in the C arm for a time significantly greater than in the B arm (blocked) (*p* < 0.01). In the case of the C57BL/6 strain, mice spent a significantly greater time in the B arm than in A or C arm. The comparison of the time that mice spent in the B arm between both strains showed that in the strain C57BL/6 it was significantly higher (*p* < 0.001) (Figure 6). In both strains one animal did not show exploratory activity during the first 2 to 3 min of the task and was rejected (91% acceptance).

### 3.2. Object Recognition Task

**Figure 7:**
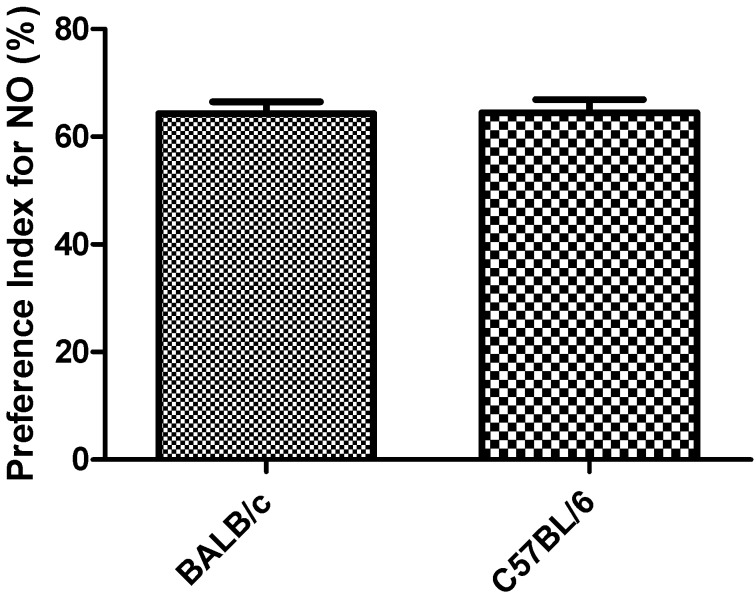
Response to the object recognition task in strains BALB/c and C57BL/6 mice by preference Index for NO-Novel Object. Data shows mean ± standard deviations for groups.

PI in the object recognition task did not showed significantly differences between the two strains (Figure 7). One mouse of each strain did not show exploratory activity during the first 2 to 3 min of the task and was rejected (91% acceptance).

### 3.3. Passive Avoidance Task

**Figure 8:**
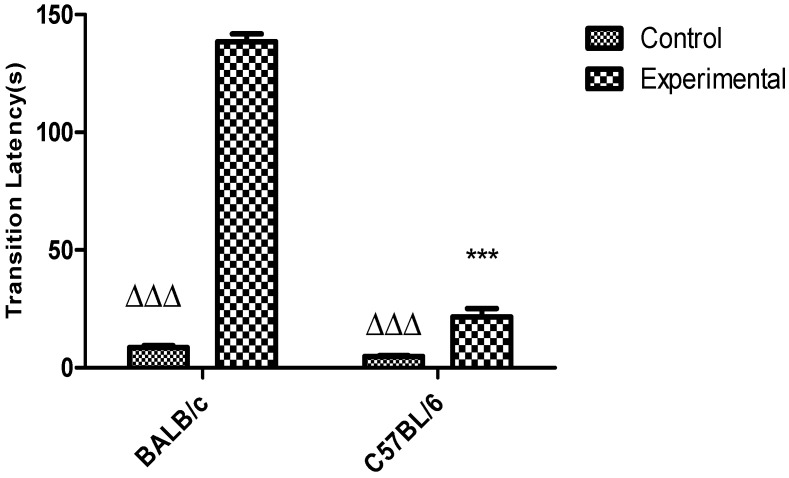
Response to the passive avoidance task in strains BALB/c and C57BL/6J. Data shows mean ± standard deviations for groups. * Differences between both strains for experimental groups. ∆ Differences between experimental and control groups (not electric shock) for each strain. A two-way ANOVA was used and *p* < 0.05.

Transition latency was higher in the experimental groups compared to the control group (not electric shock) (*p* < 0.001). (Figure 8). On the other hand, the response of the experimental group of the C57BL/6 strain was significantly shorter than for the BALB/c strain (*p* < 0.001). Only one mouse was rejected for the BALB/c strain after the period of habituation. This animal delayed more than 180 s without going to the DC to receive the electric shock.

## 4. Discussion

Behavioral tasks to evaluate cognitive function in laboratory animals are tools with a key role during the development of pharmaceutical formulations aimed to the treatment of neurodegenerative diseases [2,5,6]. It has been reported that C57BL/6 and BALB/c strains differ in their sensitivity to early life stress, with BALB/c being more susceptible to stress-induced behavioral impairment [17]. The greater sensitivity to early life stress in BALB/c strain, which stimulate the exploratory behavior [13], may explain the results obtained in this work during the Y-maze tasks in relation to the higher number of alternation for this strain. The number of incorrect entries to the arm, which was lower for the C57BL/6 could be considered an advantage during the Y-maze task with the three open arms for this strain (Figure 4). The lower number of incorrect entries in C57BL/6 strain also explains the result obtained in relation to the spontaneous alternation that showed no differences between the two strains (Figure 5), although the mice of the BALB/c strain showed a higher number of alternations (Figure 4). These results indicated that mice of C57BL/6 strain participated more slowly in the alternation task because they spent more time to elucidate which was the last arm visited. On the other hand, the spontaneous alternation results obtained for both strains of mice in this work are similar to those obtained by Hiramatsu et al. [22], when they used this task for IDD mice and obtained a spontaneous alternation of 70%.

To analyze the usefulness of the Y-maze task with one arm blocked during any experimental design with animal models to evaluate the cognitive function, it would be expected that the time spent in the novel arm (arm blocked), should be significantly higher than the time spent in the other two arms [23]. The behavior for the BALB/c strain for the Y-maze task with an arm blocked, under the experimental conditions used in this work, demonstrated the non-effectiveness of this task for this strain of animals (Figure 6). This task was repeated for the BALB/c strain by shortening the returned time for mice to the Y-maze with the three arms free (only 20 min). Under these experimental conditions the mice also did not spend more time in the blocked arm (results not shown). We suppose that these results have not been influenced by the environmental conditions. Both strains were evaluated under the same conditions of noise and lighting and neither visual cues (intra or extra maze) were used to stimulate the higher preference for the novel arm to the C57BL/6 strain. On the other hand, influence in visual acuity and olfactory response differences between both strains were also ruled out, as it has been demonstrated by Brown et al. [24]. In relation to the results obtained about the time spent in the novel arm for C57BL/6 strain, it is greater than the reported for Liang-Wen et al. [22], when after 1 h the mice returned to the Y-maze task with the three open arms.

The results obtained in the object recognition task in relation to PI for NO (above 50%), for the two strains (Figure 7), indicates novel object preference [21]. Objects used in the task and the rest of the experimental conditions were able to activate the memory mechanism which allowed the animals to identify the objects by their shape, activating their exploratory behavior to the NO. On the other hand, PI for the NO in C57BL/6 strain are in agreement with the results obtained by Liang-Wen et al. [23], under an experimental design similar to the one used in this work. These results also demonstrate the biological differences between both strains since each one was motivated by different objects (plastic cap of volumetric flask for BALB/c and plastic cylinder for C57BL/6 strain).

The passive avoidance task induces in the rodent a conflict between the innate preference for the dark areas and its relationship with an aversive stimulus, which in this case is the electric discharge that mice receive in the DC [3]. The results obtained in the current study in relation to transition latency also corroborate the biological differences between the two strains of mice (Figure 8). For C57BL/6 strain these results are similar to those obtained by Laugero et al. [10], a study in which they used a stimulation of 0.5 mA during 2 s and transition latency to the DC was for about 25 s in healthy animals. For C57BL/6 mice the response in relation to latency transition could be increased by the evaluation of the passive avoidance task under other conditions as the reduction of the reduction time interval in returning the animal to the task after having received the electrical shock, or by the increase of intensity and duration of the electrical stimulus. The results obtained for the latency transition between these strains can be explained by the differences that exist in fear memory. BALB/c mice showed a stronger fear memory than C57BL/6 mice that is explained by their differences in the response to corticosterone [11,16]. In addition, these results may also suggest that BALB/c strain is more sensitive to the pain induced by electric discharges than the strain C57BL/6.

We consider that the percentage of valid animals, whose lower values were found between 83 and 91% (corresponds to one or two animals rejected from a sample of 12), are acceptable due to the complexity of animal behavior. In addition, during the execution of these behavioral tasks the animals were handled many times and this situation increased the daily stress in laboratory animals. The differences between the numbers of invalid animals of both strains can be also justified by the differences shown between these two strains in behavioral studies related to anxiety, fear and stress [11,12,13,16,17,18,19,24].

The present work is focused in the behavioral consequences of genetic differences in the two inbred mice, BALB/c and C57BL/6. Cognitive function tasks are widely used both, in the development of animal models of neurodegenerative diseases and during the development of new pharmaceutical formulation. This study corroborates the behavioral differences between laboratory mice strains and it allows gaining knowledge about mice baseline behavior and their response during the experimental procedures, which are due to the treatment, genetic influence, procedural differences, genetic background variance, or any combination of these elements.

## Figures and Tables

**Table 1:** Previous studies comparing C57BL/6 and BALB/c mice strain in relation to differences in behavioral performance.

Aim	Reference
Inbred mouse strain differences in the establishment of long-term fear memory	Balogh et al. [11]
Emotion and cognition in high and low stress	Brinks et al. [12]
Different effect of chronic stress on learning and memory	Palumbo et al. [13]
Strain specific fear behavior and glucocorticoid response to aversive events	Brinks et al. [16]
Strain-Specific Cognitive Deficits in Adult Mice Exposed to Early Life Stress	Mehta and Schmauss [17]
Effects of conditioned fear in adult mice following postnatal exposure to chlorpyrifos	Oriel and Kofman [18]
Variability in empathic fear response among inbred strains of mice	Keum et al. [19]
